# Effectiveness of an ERAS-based exercise-nutrition management model in enhancing postoperative recovery for thoracoscopic radical resection of lung cancer: A randomized controlled trial

**DOI:** 10.1097/MD.0000000000037667

**Published:** 2024-04-12

**Authors:** Lingqiao Huang, Yingying Hu, Junxian Chen

**Affiliations:** a Department of Surgery, Jinhua Hospital of Chinese Medicine, Jinhua, Zhejiang, China; b Department of Nutrition, Jinhua Hospital of Traditional Chinese Medicine, Jinhua, Zhejiang, China.

**Keywords:** exercise, lung neoplasms, nutritional status, perioperative period, rehabilitation, thoracoscopy

## Abstract

**Background:**

To analyze the effect of an exercise-nutrition management model based on the Enhanced Recovery After Surgery (ERAS) concept on patients undergoing thoracoscopic radical surgery for lung cancer.

**Methods:**

From June 2019 to December 2022, 85 lung cancer patients who underwent thoracoscopic radical lung cancer surgery were randomly divided into 2 groups. The control group, consisting of 42 patients, received routine nursing care during the perioperative period. The study group, comprising 43 patients, implemented an exercise-nutrition management model based on the ERAS concept during the perioperative period. We compared general data, perioperative indicators, compliance, and complications between the 2 groups. Additionally, we assessed the nutritional status using the patient-generated subjective global assessment (PG-SGA), albumin (ALB), prealbumin (PA), and hemoglobin (Hb), as well as lung function, including forced expiratory volume in the first second (FEV1) and maximum voluntary ventilation (MVV), in the patient population following the Piper intervention.

**Results:**

In the study group, the times to first defecation and getting out of bed, the duration of thoracic drainage tube indwelling, and the length of hospital stay were shorter than those in the control group. The VAS scores on the 2nd and 3rd postoperative days were lower in the study group than in the control group (*P* < .05). Medication compliance was higher in the study group compared to the control group (*P* < .05). Post-intervention, the PG-SGA scores in the study group were lower, while PA, ALB, and Hb levels were higher than those in the control group (*P* < .05). The MVV, FEV1, and FVC values were higher in the study group than in the control group after the intervention (*P* < .05). The PFS and mMRC scores were lower in the study group compared to the control group after the intervention, and the QLQ-C30 scores were higher (*P* < .05). The incidence of complications was 6.98% in the study group, which was not significantly different from 11.90% in the control group (*P* > .05).

**Conclusion:**

The exercise-nutrition management model, based on the ERAS concept, exhibits significant perioperative effects in patients undergoing thoracoscopic radical resection of lung cancer, improving their nutritional status and reducing complications.

## 1. Introduction

At present, chronic diseases such as cardiovascular diseases, diabetes, chronic respiratory diseases, and cancer have become major global health challenges. According to a report by the World Health Organization, chronic diseases are the leading cause of death worldwide, accounting for 63% of all global deaths.^[1]^ Unhealthy dietary habits, characterized by high fat, high sugar, and low fiber intake, are considered one of the key factors contributing to the rising incidence of chronic diseases. In contrast, diets rich in natural compounds, such as fruits, vegetables, and whole grains, have demonstrated potential in reducing the risk of chronic diseases.^[2,3]^ Moreover, regular physical training not only improves overall health and enhances cardiovascular function and insulin sensitivity but also reduces the risk of chronic diseases by decreasing inflammation and oxidative stress.^[4,5]^

In the field of cancer management, the role of exercise-nutrition management models is increasingly recognized. Exercise is considered a potent intervention that can play a significant role in cancer prevention and rehabilitation. Regular physical activity can not only reduce the incidence of certain types of cancer, such as lung cancer but also improve the survival rates and quality of life of cancer patients.^[6,7]^ Nutrition management is also a crucial component of cancer treatment, and appropriate nutritional support can help patients maintain the necessary energy intake. Studies have shown that an intervention model combining nutrition and physical exercise is most effective in the pre-cachexia stage and can also enhance immunity, thereby supporting the rehabilitation process of cancer patients.^[8,9]^

In summary, integrating exercise and nutrition management into a comprehensive intervention model is essential for reducing cancer risk and promoting the rehabilitation of cancer patients. The concept of Enhanced Recovery After Surgery (ERAS) offers advantages in optimizing the perioperative pathway for patients, accelerating the postoperative recovery process, and reducing the risk of psychophysiological complications.^[10]^ Previous studies have indicated the benefits of the ERAS concept in the recovery process after radical lung cancer surgery, but there is limited information available to assist patients in managing their nutrition and exercise.^[11,12]^ In light of this, the present study will explore the impact of an exercise-nutrition management model on the nutrition, recovery, and quality of life of patients following thoracoscopic radical resection for lung cancer, aiming to provide new insights and evidence for the comprehensive management of cancer patients.

## 2. Materials and methods

### 2.1. General information

This study received approval from the Ethics Committee of Jinhua Hospital of Traditional Chinese Medicine, Zhejiang Province. We selected 85 lung cancer patients who underwent thoracoscopic radical resection at our hospital between June 2019 and December 2022. The cohort comprised 48 males and 37 females, ranging in age from 42 to 81 years, with an average age of 58.98 ± 5.28 years. The average body mass index was 21.38 ± 1.41 kg/m^2. Regarding the pathological types, there were 41 cases of squamous cell carcinoma and 44 cases of adenocarcinoma. In terms of TNM staging, 63 patients were in stage I, and 22 were in stage II.

### 2.2. Inclusion and exclusion criteria

Inclusion criteria: Patients must meet the diagnostic criteria for lung cancer as outlined in reference,^[13]^ be eligible for thoracoscopic radical resection of lung cancer as confirmed by fiberoptic bronchoscopic biopsy, have a tumor with the longest diameter of <5 cm, have no history of radiotherapy, chemotherapy, or chest surgery, and must voluntarily sign the informed consent form. Exclusion criteria: Patients with spinal cord injuries, craniocerebral trauma, or those requiring long-term bed rest; those with local peripheral neuropathy or central nervous system diseases; patients with coagulation dysfunction; individuals undergoing long-term treatment with immunosuppressants, analgesics, hormones, or other drugs; patients with severe intrathoracic adhesions, pleural thickening or calcification, pulmonary tuberculosis, lobar hypoplasia, pulmonary interstitial disease, chronic obstructive pulmonary disease, or other significant conditions; patients requiring conversion to thoracotomy; those with other malignant tumors; patients with severe hypertension or cardiovascular disease; individuals whose imaging examinations reveal distant metastasis of the lesion; and pregnant or lactating women.

### 2.3. Method

This study is a randomized controlled trial. Before the study began, we randomly assigned participants to the control group or the study group using a computer-generated random number table. The control group received conventional perioperative care, while the study group implemented an exercise-nutrition management model based on the ERAS concept, as shown in Table 1. All intervention measures were detailed preoperatively and implemented immediately postoperatively.

**Table 1:** Two groups of nursing programs.

Contents	Control group	Study group
Preoperative health education and condition assessment	Distribute health education manuals, orally explain the content of surgery and perioperative precautions, and make preoperative preparations.	Face-to-face communication with patients, according to the ability and education level of patient adopted diversified forms such as action demonstration, video playback, WeChat mini program, etc, to educate patients and family members in stages and time; For those with negative emotions such as anxiety and concerns, implemented psychological counseling; used the comprehensive subjective self-assessment scale (PG-SGA) to evaluate the nutritional status of the patient, and the nutritionist formulated an individualized nutritional plan based on the evaluation results and combined with the patient preferences and actual condition.
Preoperative dietary guidance	Preoperative fasting for 12 h and no liquids for 8 hours	Eat a regular dinner the night before surgery, fast for 6 h, and consume 500 mL of dry vegetarian food orally 2 hours before to the operation. Patients who had diabetes consumed the same quantity of fructose orally before operation.
Preoperative functional exercise	Not emphasized	Before operation, use a respiratory function training device or blowing balloon, 2–3 times/d, 10 min/time; Abdominal or septal breathing, lip retraction breathing, 3–4 times/d, 8–10 breaths/time; Two days before surgery, guide patients to exercise bedtime urination and correct coughing methods.
Preemptive analgesia	Before skin incision, the anesthesiologist administered non-steroidal anti-inflammatory drugs according to the patient weight	Same as control group
Antibiotic use	Antibiotics from 3 d before surgery to 3–7 d after surgery	Preventive use of broad-spectrum antibiotics once on the day of surgery
Intraoperative warming	Not emphasized	The temperature of the infusion fluid was controlled at about 37°C; the chest cavity was flushed with warm saline; insulation measures such as covering with insulation blankets were used during the operation.
Infusion volume control	Open hydration strategy	Restrictive fluid regimen, infusion volume < 1500 mL
Nutrition Management	After 4–6 h, eat a small amount of liquid food, if no discomfort symptoms, then gradually switch to normal food	For patients with preoperative PG-SGA score ≥ 2 points, enteral nutrition intervention was started; for patients with preoperative PG-SGA score ≥ 2 points, parenteral nutrition was adopted 1 d after operation; 2–3 d after surgery, enteral nutrition (50%) + parenteral nutrition (50%); 4–5 d after surgery, enteral nutrition (70%) + parenteral nutrition (30%); 6–7 d after operation, enteral nutrition, TPF type enteral nutrition suspension (GYZZH20103536, Nutricia Pharmaceutical Co., Ltd.), selects Fuerkai enteral nutrition infusion pump and Infusion tube. Patients with a PG-SGA score of 0–2 were given 50–100 mL of warm water 4–6 h after surgery, and if no discomfort observed, a small amount of full fluid diet was given, and gradually switched to semi fluid and general diet
Analgesic management	Analgesic pump plus pethidine injection	48 h after surgery, the analgesic pump was continuously administered intravenously; oral non-steroidal analgesics, reduce use of opioids.
Exercise management	Take a sitting posture 24 hours after the procedure, pat the back to release sputum, and get out of bed as the patient desires.	After waking up from anesthesia, after 6 h of stable vital signs take the lower semi-recumbent position, and encourage the patient to practice coughing, deep breathing, and moving limbs; 1–3 d after surgery, perform respiratory function training, raise the head of the bed by 90°, take a sitting position, perform straight leg lifting, ankle pump exercise, knee flexion and hip raising; Follow the “Four Step Plan” of getting out of bed, and encourage patients to get out of bed.
Discharge guidance	Periodic review	Before discharge from the hospital an educational manual was issued to patients, explaining the precautions for home care for patients and their families; Follow up through WeChat platforms, phone calls, and patients were urged to return to the hospital for follow-up visits on a regular basis.

PG-SGA = patient-generated subjective global assessment.

### 2.4. Observation indicators

Perioperative Indicators: We recorded the time of the first defecation post-operation, the first time the patient got out of bed post-operation, the duration of chest drainage tube placement, the length of postoperative hospital stay, and the degree of postoperative pain. The degree of pain experienced by patients in an active state (such as turning over) on the 1st, 2nd, and 3rd days post-operation was evaluated using the Numerical Pain Rating Scale (NRS), where 0 indicates no pain and 10 indicates severe pain. Compliance: The Morisky Medication Compliance Questionnaire, developed by Morisky in 1986,^[14]^ was utilized to assess patients’ compliance behavior 3 months post-discharge. The scale has a maximum score of 8, with scores below 6 indicating poor compliance, scores between 6 and 7 indicating moderate compliance, and a score of 8 indicating good compliance. The Cronbach α coefficient of the scale is 0.717. Nutritional Status: The nutritional status of patients was assessed using the comprehensive subjective self-assessment scale (PG-SGA) before the intervention and 7 days post-intervention. The PG-SGA assesses various factors including food intake, physical function, body weight, metabolism, and symptoms related to nutritional needs. A score between 0 and 1 indicates good nutrition, scores between 2 and 8 indicate mild to moderate malnutrition, and scores of 9 or higher indicate severe malnutrition. We collected 3 mL of fasting venous blood from patients before and 7 days after the intervention, centrifuged it at 3000 rpm for 10 minutes with a centrifugal radius of 6 cm, and measured serum albumin (ALB), prealbumin (PA), and hemoglobin (Hb) levels using an automatic biochemical analyzer (manufacturer: Siemens Medical Devices Co., Ltd., model: Chemistry XPT). Lung Function: The forced vital capacity (FVC), forced expiratory volume in the first second (FEV1), and maximum voluntary ventilation (MVV) of the patients were measured before and 7 days after the intervention using a pulmonary function detector (manufacturer: Weiya’an Medical Instrument Co., Ltd., model: MasterScreen). Fatigue, Quality of Life, and Dyspnea: The Piper Fatigue Scale (PFS) consists of 22 items across 4 dimensions: emotional, behavioral, cognitive, and physical, with each item scored from 0 to 10. The total score is the average of the 22 items, with higher scores indicating more severe fatigue. The Quality of Life Scale (QLQ-C30) comprises 5 domains: cognitive function, social function, emotional function, physical function, and overall health, with a total score of 100 points, where higher scores indicate better quality of life. The modified British Medical Research Council Dyspnea Index Scale (mMRC) is scored from 0 to 4, with higher scores indicating more severe dyspnea symptoms. The assessments for these 3 scales were conducted before and 3 months after the intervention.

### 2.5. Statistical methods

The data was analyzed using SPSS 24.0 software. The *t* test was used for measurement data expressed as $\bar{x}\pm s$, and the count data were expressed as a percentage utilizing *χ*^2^ test. *P* < .05 indicated that the difference was statistically significant.

## 3. Results

### 3.1. General information

The gender, age, body mass index, tumor diameter, pathological type, and TNM stage of the study group were compared with those of the control group (*P* > .05). See Table 2.

**Table 2:** Comparison of general information between the 2 groups (n, χ̄±s).

Group	Number of cases	Male/Female	Age (yr)	BMI (kg/m^2^)	Tumor diameter (cm)	Pathological type	TNM
						Squamous cell carcinoma/adenocarcinoma	I/II
Control group	42	23/19	58.65 ± 5.11	21.68 ± 1.38	2.49 ± 0.85	21/21	30/12
Study group	43	25/18	59.35 ± 5.84	21.23 ± 1.18	2.85 ± 0.84	20/23	33/10
*χ* ^2^		0.099	0.588	1.617	1.964	0.104	0.313
*P*		.754	.558	.120	.053	.748	.576

BMI = body mass index.

### 3.2. Perioperative indicators

**Figure 1.**
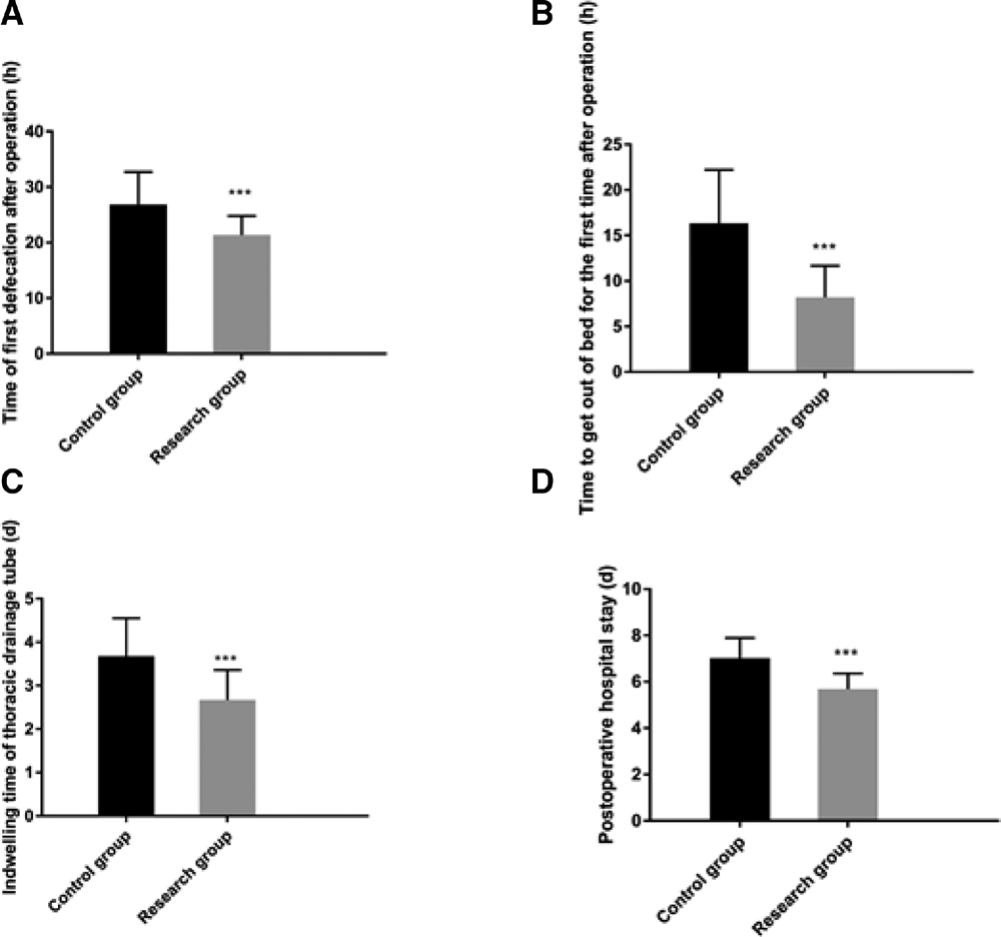
Comparison of perioperative indicators between the 2 groups. Note: Compared with the control group, ^***^P < .001.

**Figure 2.**
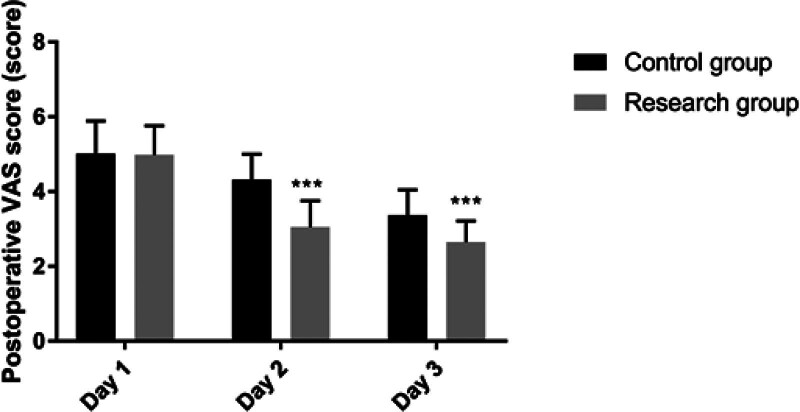
Comparison of VAS scores at different time points after operation between the 2 groups. Note: Compared with the control group, ^***^P < .001.

The study group had shorter postoperative first bowel movement time, the first time to get out of bed after operation, thoracic drainage tube retention time, and postoperative hospitalization time compared to the control group. The VAS scores on the 2nd and 3rd postoperative days were lower than the control group (*P* < .05). See Figures 1 and 2.

### 3.3. Medication compliance

The medication compliance of the study group was better than that of the control group (*P* < .05). See Table 3.

**Table 3:** Comparison of medication compliance between the 2 groups n (%).

Group	Number of cases	Complete compliance	Partial compliance	Noncompliance
Control group	42	10 (23.81)	24 (57.14)	8 (19.05)
Study group	43	21 (48.83)	20 (46.51)	2 (4.65)
*Z*		2.752		
*P*		.006		

### 3.4. Nutritional status

**Figure 3.**
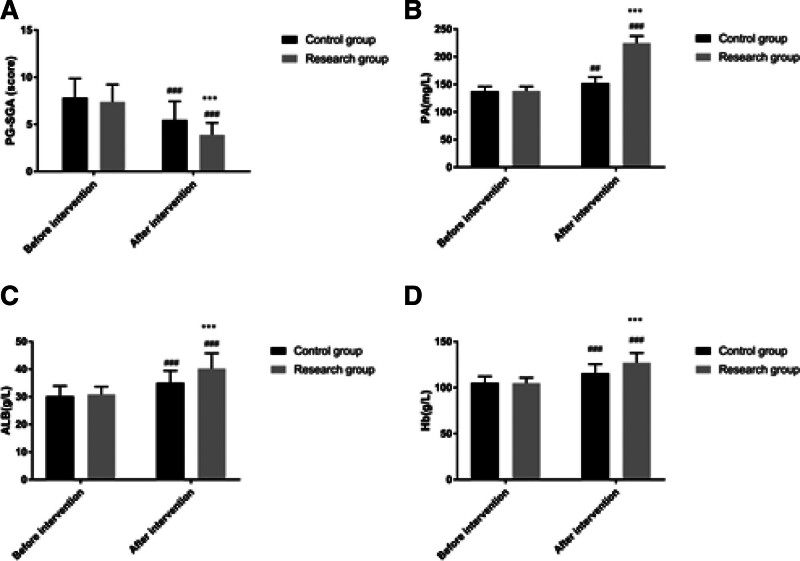
Comparison of nutritional status between the 2 groups. Note: Compared with this group before intervention, ^##^*P* < .01, ^###^*P* < .001; compared with the control group, ^***^P < .001.

There was no statistically significant difference in PG-SGA, PA, ALB, and Hb between the study group and the control group before intervention (*P* > .05) but after intervention, the PG-SGA score of the research group was lower than that of the control group, while PA, ALB, and Hb were higher than those of the control group (*P* < .05) as shown in Figure 3.

### 3.5. Lung function

**Figure 4.**
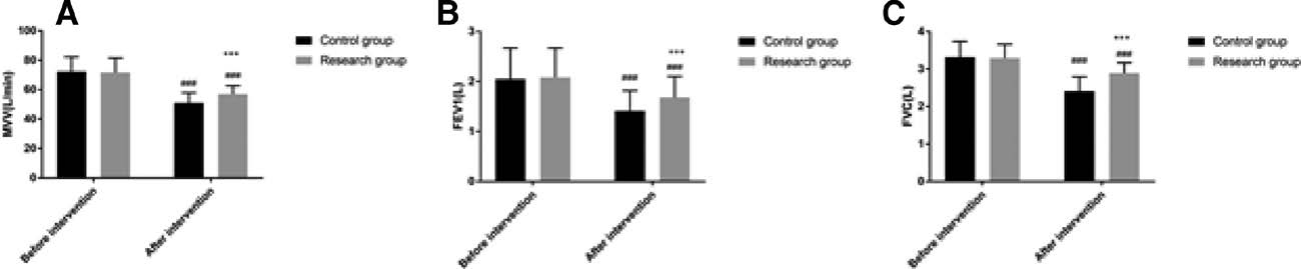
Comparison of lung function between the 2 groups. Note: Compared with this group before intervention, ^###^*P* < .001; compared with the control group, ^***^P < .001.

Before the intervention, the MVV, FEV1, and FVC of the study group were not significantly different from those of the control group (*P* > .05) but after the intervention, the MVV, FEV1, and FVC of the study group were higher than those of the control group (*P* < .05). See Figure 4.

### 3.6. Fatigue, quality of life, dyspnea

**Figure 5.**
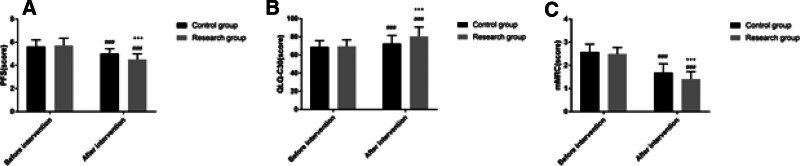
Comparison of fatigue, quality of life, and dyspnea between the 2 groups. Note: Compared with this group before intervention, ^###^*P* < .001; compared with the control group, ^***^P < .001.

Before the intervention, the PFS, QLQ-C30, and mMRC scores of the study group were not significantly different from those of the control group (*P* > .05) and after the intervention, the PFS, mMRC scores of the study group were lower than those of the control group, and the QLQ-C30 scores were higher than those of the control group (*P* < .05) as shown in Figure 5.

### 3.7. Complication

The incidence of complications in the study group (6.98%) was higher than that in the control group (11.90%) (*P* > .05). See Table 4.

**Table 4:** Comparison of complications between the 2 groups n (%).

Group	Number of cases	Lung infection	Pleural effusion	Arrhythmia	Atelectasis	Total
Control group	42	2 (4.76)	2 (4.76)	0 (0.00)	0 (0.00)	5 (11.90)
Study group	43	0 (0.00)	1 (2.33)	1 (2.33)	1 (2.33)	3 (6.98)
*χ* ^2^						0.165
*P*						.684

## 4. Discussion

In this study, we employed an exercise-nutrition management model based on the ERAS concept to explore its effectiveness in the perioperative management of patients undergoing thoracoscopic radical resection for lung cancer. Traditional postoperative care often focuses on oral education, which may not adequately meet the needs for patient self-awareness cultivation and psychophysical health adjustment. The ERAS concept, as a comprehensive preoperative and postoperative management strategy, has been proven to significantly improve the postoperative recovery process, reduce complications, alleviate pain, relieve psychological and physiological stress, and shorten hospital stays, thus accelerating the postoperative recovery process.^[15,16]^ In the field of cancer management, the role of exercise-nutrition management models is increasingly recognized. Not only can it improve overall health, but it can also reduce the risk of chronic diseases by decreasing inflammation and oxidative stress.^[4,5]^

Firstly, our study found that patients managed under the ERAS protocol had significantly shorter times to first postoperative bowel movement, first time out of bed, thoracic drainage tube retention, and length of postoperative hospital stay, as well as lower VAS scores on the 2nd and 3rd postoperative days compared to the control group. This is consistent with the findings of D’Andrilli et al, who reported that ERAS-based nursing management could effectively shorten the postoperative hospital stay and alleviate pain for patients undergoing thoracoscopic lobectomy.^[17]^ Moreover, studies by Draeger and others, as well as Larson DW, also support the effectiveness of the ERAS concept in shortening postoperative hospital stays and accelerating the recovery of postoperative organ function, with the ERAS group showing clear advantages over the routine care group in terms of readmission rates, length of hospital stay, and accelerated recovery of postoperative organ function, aligning with the findings of this study.^[18,19]^ The reasons may be attributed to: employing diverse health education methods preoperatively, educating patients and their families in stages and over time, which can correct misconceptions of patients and family members, reduce adverse psychological states before surgery, and improve medical compliance behaviors; shortening preoperative fasting time to alleviate preoperative thirst and hunger, prevent enhanced catabolism, accelerate intestinal peristalsis, and shorten postoperative gastrointestinal recovery time; intraoperative multimodal warming measures to prevent the physiological stress response caused by hypothermia and reduce catabolism; postoperative restriction of infusion volume and fluid replacement to maintain physiological balance and prevent complications caused by excessive circulatory load^[20,21]^; postoperative multimodal analgesic management to reduce sympathetic nerve excitability, block sympathetic nerves, lessen pain intensity, and alleviate stress responses. Secondly, by employing diversified health education methods and a short-term preoperative fasting strategy, our study effectively alleviated patients’ adverse psychological states before surgery and improved medical compliance. This finding highlights the importance of patient education and psychological support in the ERAS concept, aiding patients in better preparing for the preoperative and postoperative recovery process. Furthermore, the exercise-nutrition management model based on the ERAS concept improved postoperative nutritional status and lung function in our study. In particular, the strategy of combining enteral and parenteral nutrition provided essential nutritional support for patients, facilitating intestinal recovery, enhancing immunity, and improving lung function.^[22–24]^ This is supported by the reduction in PG-SGA scores and increases in PA, ALB, and Hb levels, underscoring the significance of nutritional interventions in promoting postoperative recovery.

In conclusion, the exercise-nutrition management model based on the ERAS concept is effective in the perioperative period for patients undergoing thoracoscopic radical resection for lung cancer. It can improve patients’ nutritional status, accelerate postoperative rehabilitation, enhance quality of life and lung function, reduce dyspnea, and decrease complications. Despite the positive outcomes of this study, we acknowledge its limitations, such as the relatively small sample size and all participants being from the same medical institution, which may limit the generalizability of our results. Future research should consider validation with a larger sample size across broader geographical areas and different medical settings. Additionally, the short follow-up period might not fully capture the long-term recovery process and sustainability of intervention effects. Future studies should extend the follow-up duration to assess long-term effects and potential delayed impacts. Furthermore, the exercise-nutrition management model based on the ERAS concept, with its inherent complexity and specificity, may require specialized training and resources, which should be considered when generalizing the study findings to resource-limited settings. Therefore, future research will address these study limitations to more comprehensively evaluate the long-term effects and potential benefits of the ERAS concept in postoperative management for lung cancer patients.

## Author contributions

**Conceptualization:** Lingqiao Huang, Yingying Hu, Junxian Chen.

**Data curation:** Lingqiao Huang, Yingying Hu.

**Formal analysis:** Lingqiao Huang, Yingying Hu.

**Investigation:** Yingying Hu, Junxian Chen.

**Supervision:** Junxian Chen.

**Visualization:** Junxian Chen.

**Writing − original draft:** Lingqiao Huang.

**Writing − review & editing:** Lingqiao Huang, Yingying Hu.
